# Causal Effects of Sleep Quality on Primary Headache and the Mediation via Gut Microbiota: A Mendelian Randomization Study

**DOI:** 10.1002/brb3.70129

**Published:** 2024-11-28

**Authors:** Huanghong Zhao, Dongsheng Guan, Zhen Ma, Minghui Yang, Ning Dong, Jian Guo

**Affiliations:** ^1^ Department of Brain Disease Henan Provincial Hospital of Traditional Chinese Medicine Zhengzhou China; ^2^ The First Affiliated Hospital of Henan University of Traditional Chinese Medicine Zhengzhou China

**Keywords:** gut microbiota, mediation analysis, Mendelian randomization, primary headaches, sleep quality

## Abstract

**Background:**

Previous studies have shown that sleep quality plays an essential role in primary headaches to varying degrees. However, it is unclear precisely whether gut microbiota plays a critical role in mediating changes in sleep quality and affecting primary headaches.

**Methods:**

We utilized Mendelian randomization (MR) to examine the causal relationships between sleep quality and primary headaches. The data encompass eight sleep traits (staying asleep during periods of anxiety, trouble falling asleep, daytime dozing, sleep apnea syndrome, oversleeping, undersleeping, snoring, and sleeplessness). The primary statistical method employed was inverse variance weighting. Eventually, we explored whether gut microbiota mediate the relationship between sleep quality and primary headaches.

**Results:**

Our study found that a genetic predisposition to poor sleep quality increases the risk of primary headaches. Two‐step MR analysis revealed that the genus *Coprococcus1* mediates the causal relationship between trouble falling asleep and cluster headaches, with a mediating effect of 23.6%. These findings could inform targeted interventions and treatments for primary headaches.

**Conclusion:**

This study suggests that trouble falling asleep increases the incidence of cluster headaches mediated by gut microbiota. It highlights the crucial impact of sleep quality on primary headaches.

## Graphical abstract

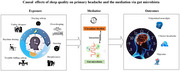

## 1 Introduction

Primary headaches, such as migraine, trigeminal neuralgia, and cluster headaches, are severe neurological conditions that significantly impair daily functioning and quality of life (Levin 2022). One of the critical factors contributing to primary headaches is circadian rhythm disruption. Studies have shown that circadian rhythm disruptions and sleep disturbances are significant triggers for primary headaches (Pellegrino et al. 2018). For instance, irregular sleep patterns and altered sleep–wake cycles have been linked to increased migraine frequency and severity (Pergolizzi et al. 2020). The disruption of the body's internal clock affects hormonal regulation, inflammation, and neural activity, all of which can exacerbate headache symptoms (Sharav, Katsarava, and Charles 2017).

Increasing epidemiological evidence highlights the significant impact of sleep quality on primary headaches. Various forms of sleep disturbances, such as insomnia, sleep apnea, and narcolepsy, have been associated with an elevated risk of developing primary headaches (Suzuki et al. 2024). Furthermore, research indicates that poor sleep quality, including frequent trouble falling or staying asleep, oversleeping, and undersleeping, is strongly correlated with the occurrence and intensity of trigeminal neuralgia (Yang and Wang 2017). Individuals with insomnia are more likely to experience migraines, while those with sleep apnea are prone to cluster headaches (Lovati et al. 2017). These findings emphasize the critical role of sleep quality in managing and preventing primary headaches.

The gut microbiota, consisting of trillions of microorganisms in the human intestines, is integral to various physiological processes, including those related to the gut–brain axis (Parekh, Oldfield, and Johnson 2018). This bidirectional communication network between the gut microbiota and the brain influences mental health, sleep, and pain perception (Afzaal et al. 2022). Clinical studies have demonstrated that alterations in gut microbiota can impact sleep patterns, contributing to sleep disorders (Socała et al. 2021). Similarly, imbalances in gut microbiota have been implicated in the development of primary headaches, particularly migraines, through mechanisms involving inflammation and neural modulation (Rinninella et al. 2019). These observations suggest that gut microbiota might mediate the relationship between sleep quality and primary headaches. Targeting the gut microbiota could offer novel therapeutic approaches to manage and prevent primary headaches by improving sleep quality.

Genome‐wide association studies (GWAS) examine millions of genetic variants in an individual's genome, providing insights into complex diseases. Mendelian randomization (MR) is less affected by environmental factors and reverse causality and is a robust genetic epidemiological tool (Birney 2022). In MR studies, genetic variation is used as an instrumental variable (IV) to assess causality between exposures and outcomes and, unlike typical observational studies, it utilizes pooled estimates of exposures and outcomes from genetic databases from different sources to improve statistical power and thus enhance the assessment of causal effects between exposures and outcomes (Bowden and Holmes 2019).

In this study, we conducted a comprehensive MR analysis of the causal relationship between sleep disorders and primary headaches. Based on this analysis, we explored whether the destruction of gut microbiota is a potential pathological mechanism in this pathogenesis process. Through a strong risk assessment, we hope to provide valuable insights into the control and harm management of primary headaches.

## 2 Methods

### 2.1 Study Design

**FIGURE 1:**
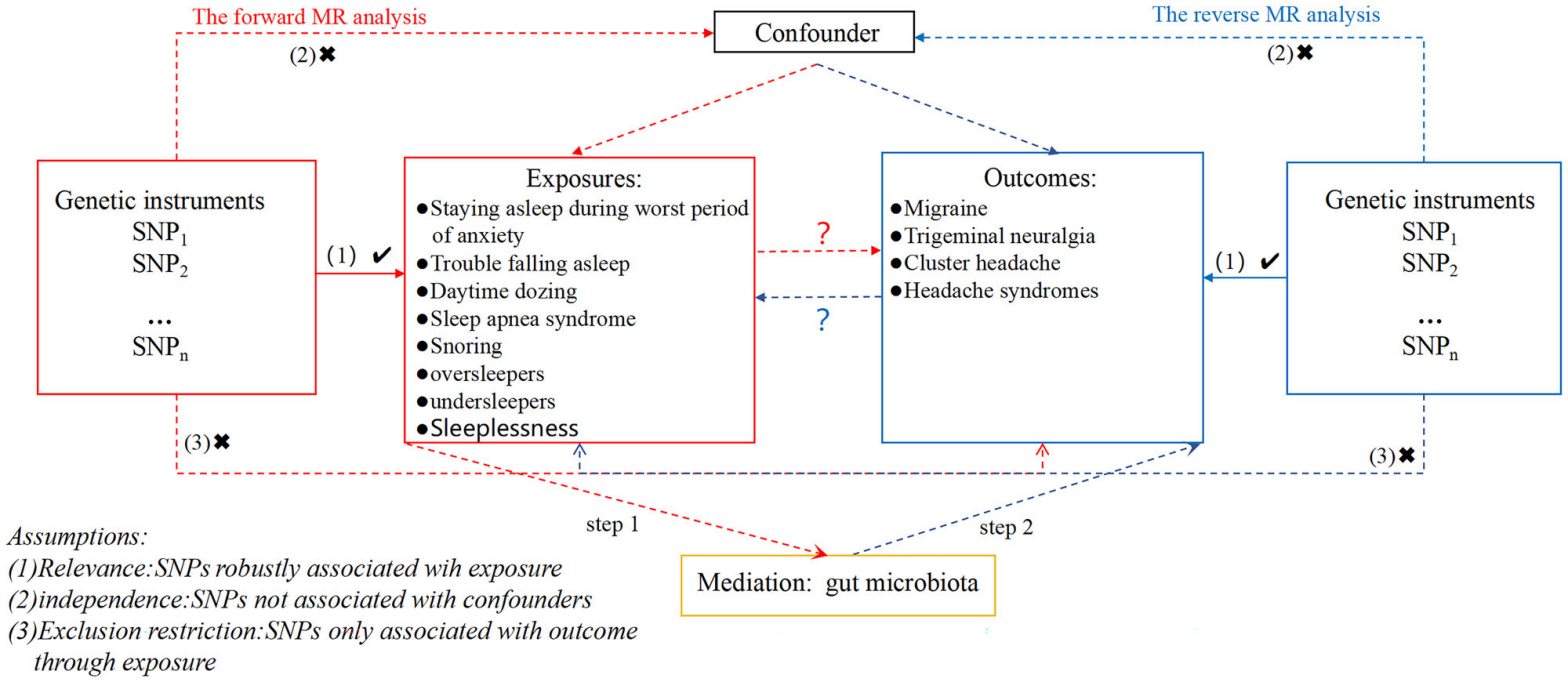
Overview of this bidirectional MR study design.

Our study provided a comprehensive flowchart illustrating the study's design in detail (Figure 1). First, we examined the causal relationship between sleep quality as exposure and primary headache as an outcome. Second, we further evaluated the correlation between exposure media (sleep quality and gut microbiota) and the correlation between media outcomes (gut microbiota and primary headache). On this basis, we assess the potential gut microbiota as the mediations in these relationships. This paper is a secondary analysis of publicly available summaries of GWAS data analyses. Ethical approval was obtained for each of the original GWAS studies, and no individual‐level data were used in this investigation, so no further ethical review board consent was required.

### 2.2 Data Source

The GWAS data on various aspects of sleep quality, including “staying asleep during the worst period of anxiety,” “trouble falling asleep,” “daytime dozing,” and “sleeplessness”: I needed less sleep than usual,“ were obtained” from the UK Biobank (https://www.ukbiobank.ac.uk). Data related to “sleep apnea syndrome,” “oversleeping,” “under sleepers,” and “snoring” were sourced from the IEU Open GWAS database (https://gwas.mrcieu.ac.uk/).

Genetic data on the gut microbiota were obtained from the MiBioGen consortium, which includes data from 24 cohorts encompassing genome‐wide genotypes and 16S fecal microbiome data for 18,340 individuals. The GWAS summary data covered 211 gut microbial taxa, including 131 genera, 35 families, 20 orders, 16 classes, and 9 species.

Data for primary headaches, including “cluster headache,” “migraine,” “trigeminal neuralgia,” “benign paroxysmal vertigo,” and “headache syndromes,” were obtained from the 8th version of the FinnGen consortium (https://r8.risteys.fnngen.fi/). Detailed information on participants for each GWAS database is provided in Table 1.

**TABLE 1:** Detailed information on included traits in the present study.

Traits	Ancestry	Consortium	Sample size (cases/controls)	Data Source
Sleep quality				
Staying asleep during worst period of anxiety	European	SSGAC	35,584 (29,129/6455)	ukb‐d‐20427
Trouble falling asleep	European	NA	45,540 (34,491/11,049)	ukb‐d‐20533
Daytime dozing	European	NA	357,957	ukb‐a‐15
Sleeplessness	European	NA	336,082	ukb‐a‐13
Sleep apnea syndrome	European	NA	348,219	ebi‐a‐GCST90018696
Snoring	European	NA	178,337 (473/177,864)	ebi‐a‐GCST009761
Oversleepers	European		91,360 (10,102/81,204)	ebi‐a‐GCST006685
Undersleepers	European		110,188 (28,980/81,208)	ebi‐a‐GCST006686
Primary headaches				
Migraine	European	FinnGen	333,783 (20,908/312,803)	https://r8.finngen.fi/
Trigeminal neuralgia	European	FinnGen	362,315 (1777/360,538)	https://r8.finngen.fi/
Cluster headache	European	FinnGen	314,017 (1214/312,803)	https://r8.finngen.fi/
Headache syndromes	European	FinnGen	314,017 (1214/312,803)	https://r8.finngen.fi/

### 2.3 IVs Selection

Our study identified single‐nucleotide polymorphisms (SNPs) significantly linked to sleep quality with a significance level below 5 × 10^−8^. We set a stringent SNP selection threshold at 5 × 10^−8^ to fully leverage the genetic instruments available. We excluded any SNPs that exhibited linkage disequilibrium (LD) with an LD cutoff of *r* ^2^ < 0.001 and isolated by more than 10,000 kb from our analysis. Furthermore, we removed SNPs that met these criteria to ensure robustness in our findings. In the MR framework, it is essential to ensure that the effects of SNPs on exposure align with the impacts of associated alleles on the outcomes (Widding‐Havneraas and Zachrisson 2022). To maintain data integrity, alleles were removed post‐matching.

### 2.4 MR Analysis

#### 2.4.1 Primary Analysis

As indicated in Figure 1, we employ two‐sample MR analyses to evaluate the causal impact of sleep quality on primary headaches. The Wald ratios test was used within the inverse variance weighted (IVW) approach as the primary statistical method for variables with a single IV. The 95% confidence intervals (CIs) and odds ratios (ORs) of the MR outcomes were reported. A *p* value lower than 0.05 in the IVW method was considered statistically significant (Burgess et al. 2017).

#### 2.4.2 Sensitivity Analysis

In each phase of our study, we utilized R (version 4.3.2) as our statistical analysis software. The MR analyses were performed with the TwoSampleMR package in R. We conducted the Cochran's *Q* test to assess heterogeneity among SNPs (Cohen et al. 2015). Scatter plots were generated to display the relationships between SNPs and exposure, highlighting the outcomes of the MR study.

In the leave‐one‐out analysis, each SNP was systematically excluded one at a time to test its influence on the outcome. Subsequently, the IVW method was applied to the remaining SNPs to evaluate their potential impact on our predictions (Gurung et al. 2020). We also explored the possibility of horizontal pleiotropy using MR‐PRESSO and MR‐Egger regression techniques. MR‐PRESSO identified significant outliers, and their removal was crucial for adjusting the effects related to horizontal pleiotropy (Plana, Pérez, and Zamora 2021).

#### 2.4.3 Mediation Analysis

We conducted a two‐step MR analysis to evaluate the impact of sleep quality on primary headache patients. The indirect effect for each identified mediator (gut microbiota taxa) between sleep quality exposure and the primary headache was individually assessed. In the first step, we estimated the effect of sleep quality exposure on gut microbiota abundance (*β*_1_) (VanderWeele 2016). We estimated the causal relationship between gut microbiota abundance and primary headache (*β*_2_) in the second step (Yao et al. 2022). Eventually, according to the “coefficient product,” the mediating effect is calculated using the *β*_1_ × *β*_2_ methods, and the mediating effect is obtained by dividing the proportion of the total effect mediated by the abundance of gut microbiota by the standard error of using the delta method to obtain the mediating effect (Carter et al. 2021) (Steps 1 and 2 in Figure 1).

## 3 Results

### 3.1 Genetic Instruments

The initial group of SNPs we discovered, at a significance level of *p* < 5 × 10^−8^, was associated with staying asleep during worst period of anxiety (12 SNPs), trouble falling asleep (10 SNPs), daytime dozing (70 SNPs), sleep apnea syndrome (2 SNPs), oversleepers (22 SNPs), undersleepers (14 SNPs), snoring (141 SNPs), sleeplessness (117 SNPs), and manic/hyper symptoms: I needed less sleep than usual (7 SNPs), respectively. These 396 SNPs were subsequently selected as IVs (see Table S1). The initial group of SNPs we discovered at a *p* < 1 × 10^−5^ level was connected to 211 distinct gut microbiotas at the class, family, genus, order, and phylum stages, respectively. For the 211 gut microbiotas taxa, these 2560 SNPs were selected as IVs (see Table S2).

### 3.2 The Causal Effects of Sleep Quality on Primary Headache

**FIGURE 2:**
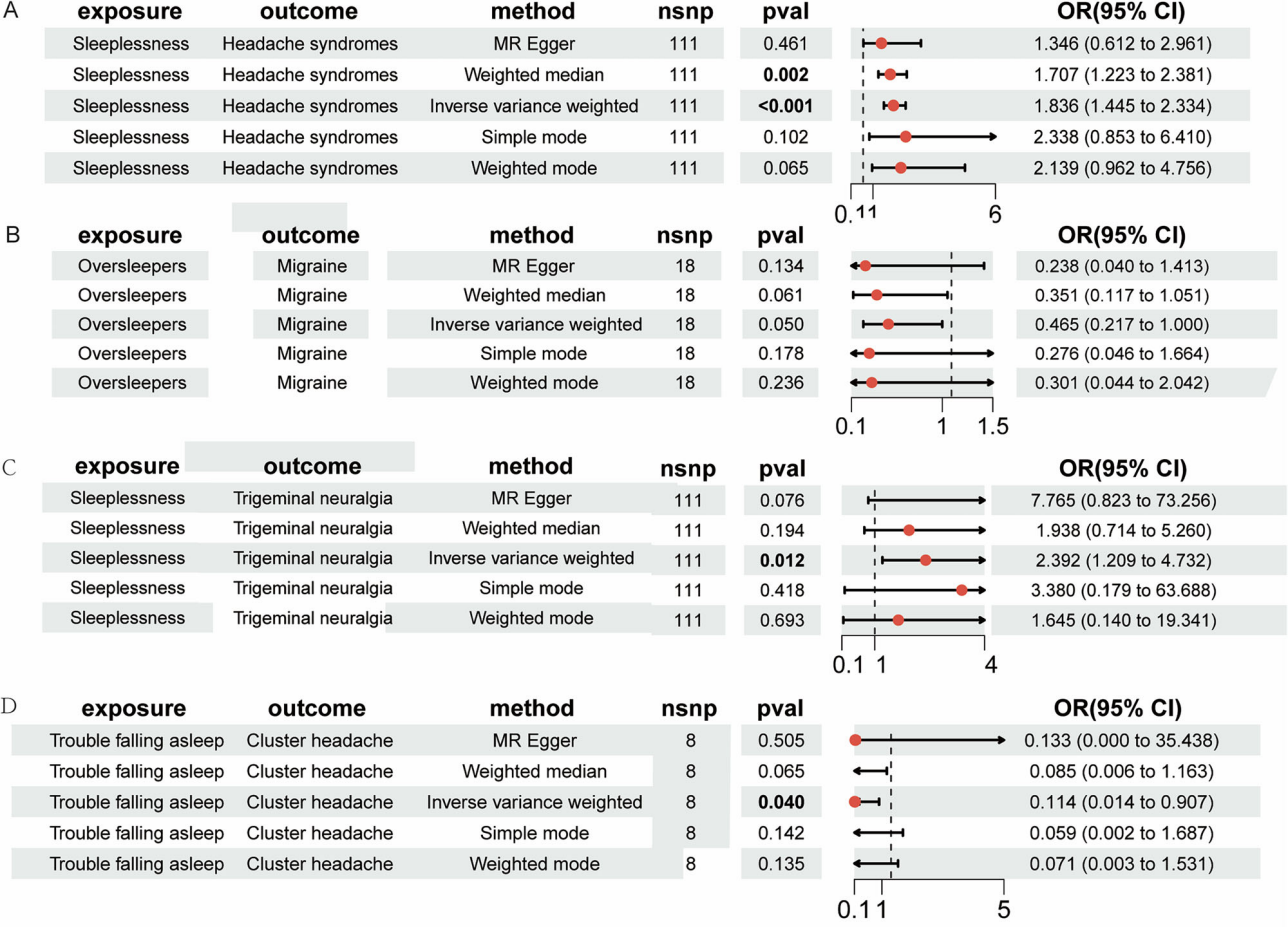
The causal effects of sleep quality on primary headache.

As illustrated in Figure 2, MR analysis reveals that sleeplessness was associated with a significant risk of headache syndromes (OR = 2.067, 95% CI = 1.15–3.697, *p* = 0.014) and trigeminal neuralgia (OR = 2.392, 95% CI = 1.209–4.732, *p* = 0.012). Oversleepers were identified as increasing the incidence of migraine (OR = 0.465, 95% CI = 0.217–1.000, *p* = 0.05), trouble falling asleep was associated with a significant risk of cluster headache (OR = 0.114, 95% CI = 0.014–0.907, *p* = 0.04).

### 3.3 The Causal Effects of Sleep Quality on Gut Microbiotas

**FIGURE 3:**
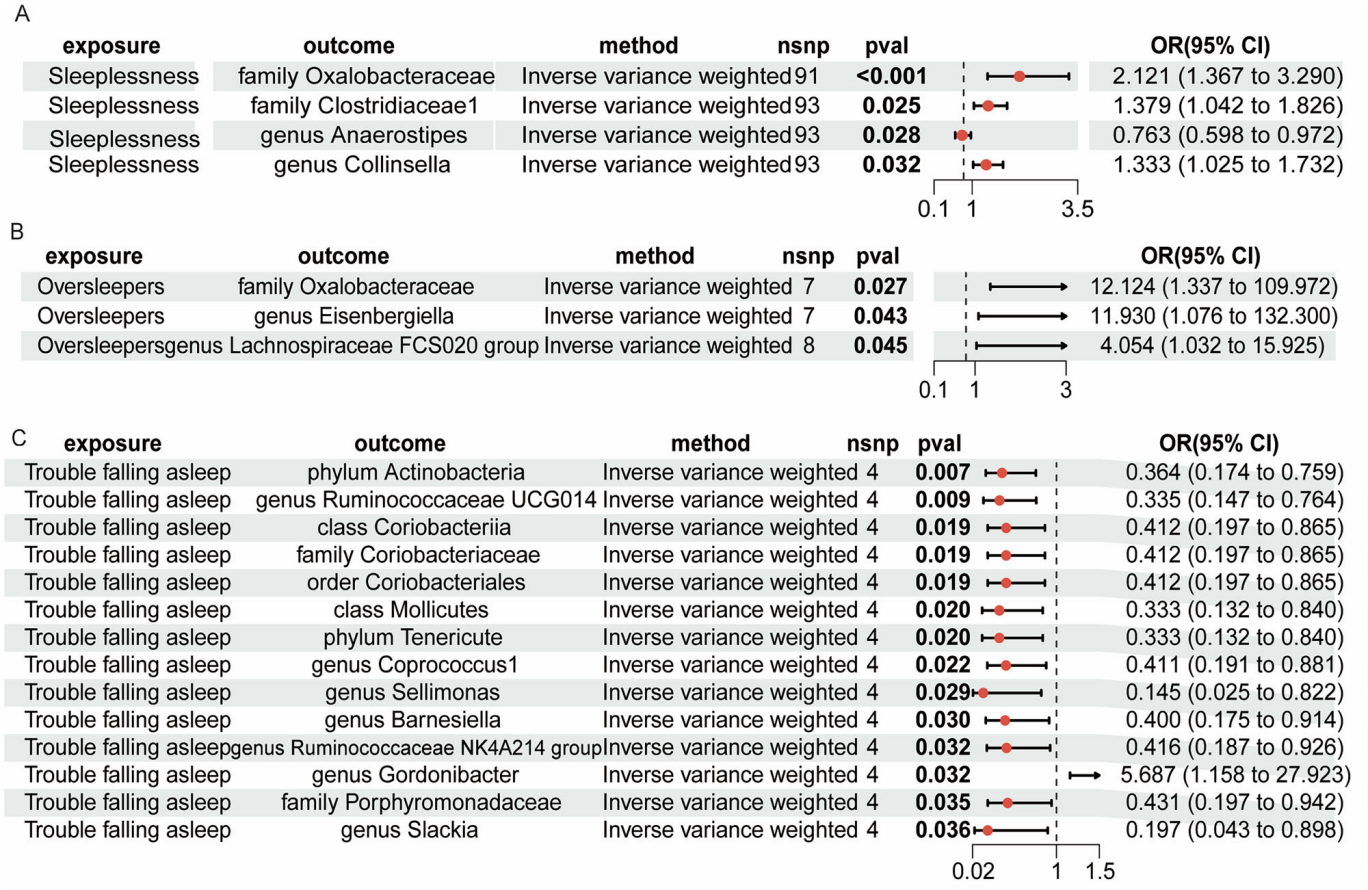
The causal effects of sleep quality on gut microbiotas.

As illustrated in Figure 3, MR analysis shows that sleeplessness was linked to a significant risk of the family Oxalobacteraceae (OR = 2.121, 95% CI = 1.367–3.290, *p* < 0.001), the family Clostridiaceae1 (OR = 1.379, 95% CI = 1.042–1.826, *p* = 0.025), and the genus *Collinsella* (OR = 1.333, 95% CI = 1.025–1.732, *p* = 0.032). Conversely, sleeplessness is associated with a lower risk of the genus *Anaerostipes* (OR = 0.763, 95% CI = 0.598–0.972, *p* = 0.028). Oversleeping was significantly associated with an increased risk of the family Oxalobacteraceae (OR = 12.124, 95% CI = 1.337–109.972, *p* = 0.027), the genus *Eisenbergiella* (OR = 11.930, 95% CI = 1.076–132.300, *p* = 0.011), and the genus *Lachnospiraceae* FCS020 group (OR = 4.054, 95% CI = 1.032–15.925, *p* = 0.045). Trouble falling asleep was significantly linked to a decreased risk of the phylum Actinobacteria (OR = 0.364, 95% CI = 0.174–0.759, *p* = 0.007) and the genus *Ruminococcaceae* UCG014 (OR = 0.335, 95% CI = 0.147–0.764, *p* = 0.009). However, trouble falling asleep was associated with a higher risk of the genus *Gordonibacter* (OR = 5.687, 95% CI = 1.580–127.923, *p* = 0.032).

### 3.4 The Causal Effects of Gut Microbiotas on Primary Headache

**FIGURE 4:**
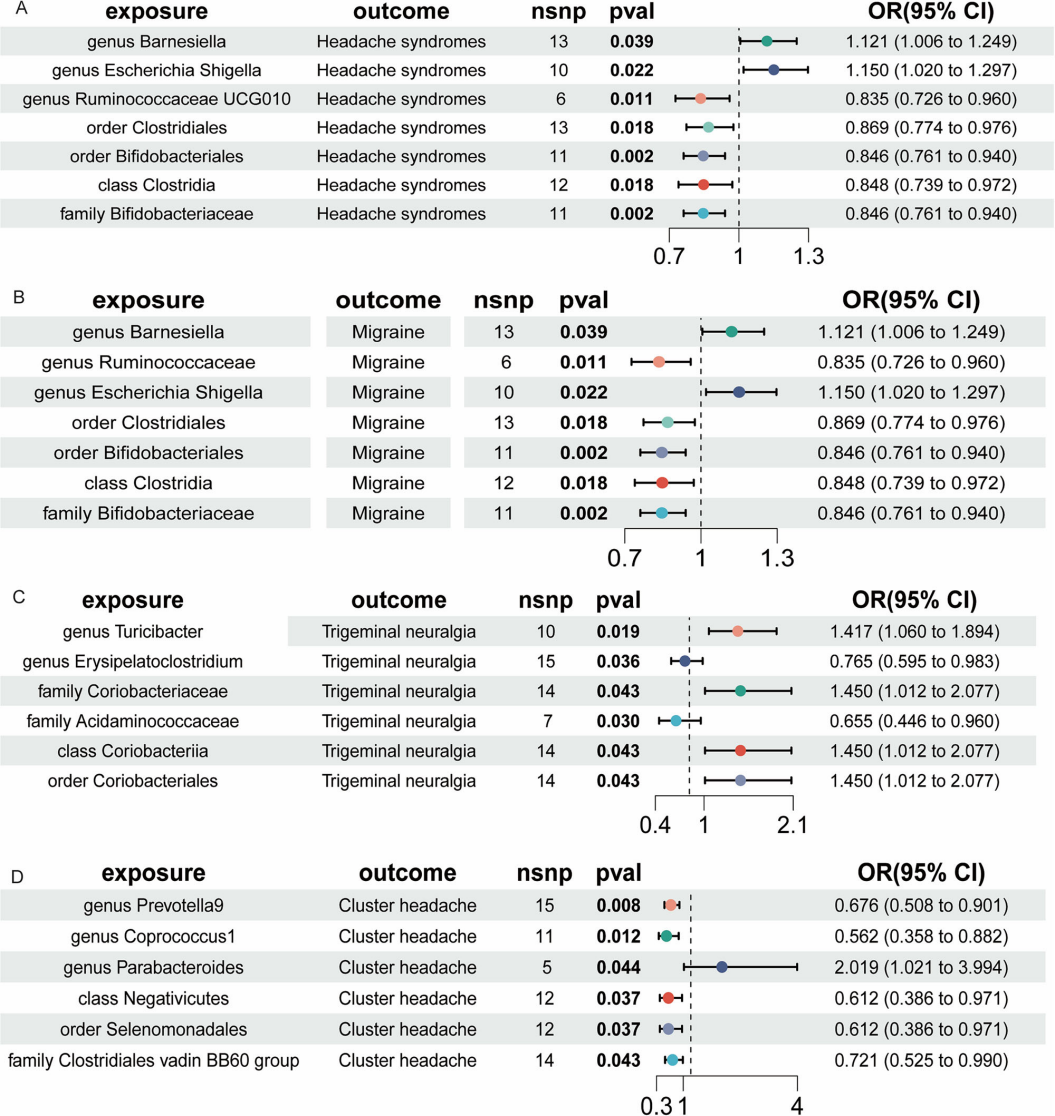
The causal effects of gut microbiotas on primary headache.

As illustrated in Figure 4, MR analysis reveals that the order Clostridiales (OR = 0.846, 95% CI = 0.761–0.940, *p* = 0.002) and the genus *Barnesiella* (OR = 0.846, 95% CI = 0.761–0.940, *p* = 0.002) Were significantly associated with a reduced risk of headache syndromes. In addition, the order Clostridiales (OR = 0.846, 95% CI = 0.761–0.940, *p* = 0.002) and the family Bifidobacteriaceae (OR = 0.846, 95% CI = 0.761–0.940, *p* = 0.002) were linked to a decreased risk of migraines. The genus *Turicibacter* (OR = 1.417, 95% CI = 1.060–1.894, *p* = 0.019) was associated with an increased risk of trigeminal neuralgia, while the family Acidaminococcaceae (OR = 0.655, 95% CI = 0.446–0.960, *p* = 0.030) was significantly linked to a lower risk of this condition. The genus *Prevotella9* (OR = 0.676, 95% CI = 0.508–0.901, *p* = 0.008) and the genus *Coprococcus1* (OR = 0.562, 95% CI = 0.358–0.882, *p* = 0.012) were significantly associated with a reduced risk of cluster headaches.

### 3.5 Sensitivity Analyses

The MR‐Egger regression intercept method results showed that genetic pleiotropy influenced them. In addition, the MR‐PRESSO test established no horizontal pleiotropy in our MR analyses. Cochran's *Q* test showed no significant heterogeneity among the studies (*p* > 0.05; see Table S3 for details).

The robustness of the MR results was further bolstered by a “leave‐one‐out” sensitivity analysis, a rigorous method that showed consistent reliability (total CIs for SNPs did not include the null hypothesis line). Scatter plots vividly described the cumulative effect of sleep quality on the risk of primary headaches. In contrast, the forest plot clearly illustrated a direct causal relationship between sleep quality and the occurrence of primary headaches (see Figure 2 for details).

### 3.6 Mediation Analysis

Utilizing the two‐step MR method for mediation analysis, we present the outcomes and the proportion of mediation attributed to specific factors. Our research findings revealed that *Coprococcus1* mediates a causal relationship between trouble falling asleep and cluster headache, with a mediating effect of *β*_1_ × *β*_2_/*α* = 23.6%. The total effect of trouble falling asleep and cluster headache was *α* = −2.175, the indirect effect of trouble falling asleep causing changes in gut microbiota was *β*_1_ = −0.89, and the indirect effect of gut microbiota causing cluster headache was *β*_2_ = 0.577 (see Table S4).

## 4 Discussion

In this study, we conducted a comprehensive MR investigation to investigate the intricate relationships between sleep quality and primary headaches, using large‐scale summary‐level statistics from GWAS. The population in these GWAS predominantly consists of individuals of European ancestry, a crucial factor that helps reduce the risk of stratification bias in our analysis. Our findings, which were of paramount importance, indicated that changes in sleep quality, such as trouble falling asleep, sleeplessness, oversleepers, and undersleepers, were associated with an increased likelihood of primary headaches. Furthermore, our mediation analysis, a novel approach, suggested that the impact of trouble falling asleep on cluster headaches was partially mediated by specific gut microbiota, pointing to the gut–brain axis as a potential pathogenic pathway for these conditions.

Primary headaches were complex and multifaceted, involving genetic predispositions, lifestyle choices, and other unmeasurable variables. The inherent differences in individual genetics influence headache development (Nyholt, Borsook, and Griffiths 2017). Despite the unpredictability and nonquantifiability of pain‐triggering factors and the need for more objective evidence in clinical settings, there still needed to be a gap in understanding the role of genetic factors in causing pain (Bron, Sutherland, and Griffiths 2021). Few clinical studies have elucidated whether sleep‐related features were precursors to primary headache attacks. Our study aims to provide objective clinical evidence by exploring whether sleep characteristics can predict primary headache attacks.

Our findings indicated that eight sleep characteristics partially promote the risk of four primary headaches (e.g., migraine, trigeminal neuralgia, cluster headache, and headaches). Poor sleep quality or disrupted sleep patterns, such as difficulty falling asleep or frequent awakenings, have consistently been linked to an increased risk of migraine attacks (Filzmoser et al. 2024). This relationship is likely due to the interaction between the brain's sleep regulation and pain processing systems, which can exacerbate the frequency and intensity of headaches (Lee et al. 2024). Moreover, studies have found that sleep quality affects primary headaches, which are usually strongly associated with specific populations. Among hospitalized patients exposed to noisy environments, a decline in sleep quality significantly increases the frequency and severity of headaches (Chen and Zhu 2024). In addition, education level and academic achievement further modulate the occurrence of headaches through their influence on sleep quality. For example, individuals with doctoral degrees are more likely to experience migraines, often due to stress, compared to those with only a bachelor's degree (Yang and Xiangming 2024). In our study, sleeplessness promotes headaches and trigeminal neuralgia, while conversely, oversleepers reduce the incidence of migraine, and trouble falling asleep reduces the incidence of cluster headaches. However, further research is needed to determine how this reduced sleep quality can reduce the incidence of migraine and cluster headaches.

There is an intrinsic biological basis for the influence of gut microbiota on primary headaches. Chronic adverse stress activates the hypothalamic–pituitary–adrenal axis, increasing levels of corticotropin‐releasing factor (CRF) and glucocorticoids, which not only causes psychological symptoms such as anxiety and depression but also activates mast cells to release proinflammatory factors, potentially triggering or exacerbating headaches; simultaneously, CRF‐induced intestinal inflammation and 5‐HT dysregulation affect the central nervous system, leading to changes in pain sensitivity and increasing susceptibility to headaches (Zhang et al. 2023). Research in rat models also shows that transplanting the gut microbiota from healthy rats into depressed rats can regulate depression‐related serum and hippocampal metabolism, thereby affecting the neurobiology and behavior of the recipients; this mechanism suggests that alterations in gut microbiota may influence the central nervous system and play a role in the pathogenesis of primary headaches (Hu et al. 2022). In our study, alterations in the abundance of a variety of gut microbiota had a significant effect on primary headache, some of which increased the occurrence of headache, while others decreased the risk of headache, for example, genus *Escherichia shigella* was able to increase the occurrence of both headache syndrome and migraine. In contrast, order Bifidobacterales and class For example, genus *E. shigella* increases the occurrence of headache syndrome and migraine. In contrast, order Bifidobacterales and class Clostridia decrease the occurrence of both.

Our mediation analysis further quantified the role of gut microbiota as a mediator in the relationship between sleep quality and primary headaches. Notably, the MR analysis revealed that specific gut bacteria, such as the genus *Coprococcus1*, significantly mediate the link between trouble falling asleep and the risk of cluster headaches, with a mediating effect of 23.6%. This finding suggested that gut health interventions offer new headache prevention and treatment possibilities. Gut bacteria like *Coprococcus1* can produce metabolites such as short‐chain fatty acids that influence inflammation and pain modulation, which are crucial in primary headaches (Gao et al. 2024). The genus *Coprococcus1* has been specifically linked to mediating the causal relationship between sleep disturbances and cluster headaches (Sun, Wang, and Kan 2024). Changes in the abundance of *Coprococcus1* significantly mediate the effect of sleep quality on headache incidence, underscoring the microbiota's role in inflammation and pain perception (Li et al. 2023). In addition, alterations in *Coprococcus1* have been associated with sleep quality, indicating that sleep disturbances can lead to shifts in gut microbiota composition, affecting systemic inflammation and pain pathways (Ferini‐Strambi, Galbiati, and Combi 2019). In conclusion, trouble falling asleep mediated by Copoccus1 increases the incidence of cluster headaches, highlighting the importance of gut health in managing sleep quality and headache disorders.

These preliminary findings, with their profound implications, underscore a bidirectional causal relationship between changes in sleep quality and primary headaches, which holds significant implications for public health and, more importantly, clinical practice. Our research, which provided a solid theoretical basis, has the potential to revolutionize interventions aimed at preventing or treating these conditions. Given the observed link between decreased sleep quality and primary headaches, interventions targeting sleep disorders could serve as a powerful tool in preventing or managing headaches (Burch, Buse, and Lipton 2019). The importance of early screening and treatment methods for primary headaches, as we propose, cannot be overstated. They are crucial for preventing related syndromes and promoting a healthy lifestyle (Stanyer et al. 2021). As our research suggests, developing good sleep habits was a valuable strategy for reducing headaches and improving gut microbiota co‐occurrence.

The main strength of our research lies in carefully using a bidirectional MR design in two samples, effectively utilizing three MR methods to mitigate bias, confusion, and reverse causality. This approach, supported by extensive sample data from GWAS, enhances the study's credibility. Furthermore, we controlled for bias by restricting participants to individuals of European ancestry. We performed MR‐Egger and MR‐PRESSO analyses, which did not find evidence of pleiotropic effects, consolidating the validity of our findings. Our exploration through two‐step MR analysis has deepened our understanding of the mechanisms involved and provided evidence for prevention strategies.

Despite its pioneering nature, our study, like any other, has certain limitations. Although we avoided sample overlap by using different public databases from Finland, UKB, and IEU Open GWAS, the inability to access detailed participant information to eliminate potential overlap may impact the robustness of our results. Our investigation was confined to the European population, underscoring the need for more diverse populations in future studies. Another limitation of this study is that gender was not taken into account. Considering that there are gender differences in sleep disorders, primary headaches, and gut microbiota, subsequent studies must explore this in more depth or differentiate between them. While our focus was on gut microbiota as mediators between sleep quality and primary headaches, other potential confounding factors, such as inflammation levels, neurotrophic factors, and epigenetics, may also play a role. Therefore, further research with more diverse populations and comprehensive datasets was not just necessary but urgent to validate and extend our findings and to continue the progress in this field.

## 5 Conclusion

In this study, we comprehensively explored whether sleep quality and gut microbiota significantly impact the development of primary headaches. The results indicate that trouble falling asleep increased the incidence of cluster headaches, partly by affecting the intestine's microbial community. These findings emphasized the importance of sleep quality on primary headaches, but further research is needed to elucidate potential pathogenic mechanisms and preventive strategies.

## Author Contributions

**Huanghong Zhao**: conceptualization. **Dongsheng Guan**: methodology. **Zhen Ma**: software. **Minghui Yang**: data curation. **Ning Dong**: investigation. **Jian Guo**: validation.

## Ethics Statement

The present study is a secondary analysis of publicly available data. Ethical approval was granted for each of the original GWAS studies. In addition, no individual‐level data were used in this study. Therefore, no new ethical review board approval was required.

## Consent

The authors have nothing to report.

## Conflicts of Interest

The authors declare no conflicts of interest.

### Peer Review

The peer review history for this article is available at https://publons.com/publon/10.1002/brb3.70129.

## Supporting information




**Table S1** Association of SNPs with sleep quality (IV Selection).

**Table S2** Association of SNPs with gut microbiotas (IV Selection).

**Table S3** Sensitivity analysis of the causal relationship between sleep quality and primary headache.

**Table S4** Mediation analysis of the causal effect of sleep quality on primary headache via gut microbiotas.

## Data Availability

The data that support the findings of this study are available on request from the corresponding author. The data are not publicly available due to privacy or ethical restrictions.
